# A *Nexus* model of cellular transition in cancer

**DOI:** 10.1186/s40659-018-0173-8

**Published:** 2018-08-07

**Authors:** Mukesh Yadav, Payal Chatterjee, Simran Tolani, Jaya Kulkarni, Meenakshi Mulye, Namrata Chauhan, Aditi Sakhi, Sakshi Gorey

**Affiliations:** 1 Department of Pharmaceutical Sciences, Softvision College and Research Institute, Vijaynagar, Indore, MP 452010 India; 2 Department of Pharmaceutical Sciences, College of Pharmacy, Western University of Health Sciences, Pomona, CA 91766 USA

**Keywords:** Cancer, The *Nexus* model, Biochemical stress, Epigenetics, Mutations, Genetics

## Abstract

The exact cause of cancer is one of the most immutable medical questions of the century. Cancer as an evolutionary disease must have a purpose and understanding the purpose is more important than decoding the cause. The model of cancer proposed herein, provides a link between the cellular biochemistry and cellular genetics of cancer evolution. We thus call this model as the “Nexus model” of cancer. The Nexus model is an effort to identify the most apparent route to the disease. We have tried to utilize existing cancer literature to identify the most plausible causes of cellular transition in cancer, where the primary cancer-causing agents (physical, chemical or biological) act as inducing factors to produce cellular impeders. These cellular impeders are further linked to the *Nexus*. The *Nexus* then generates codes for epigenetics and genetics in cancer development.

## Background

Cancer research has made an outstanding progress to identify and tackle the probable causes of the disease, which stands to be unique with respect to the organs affected and the genetic makeup of the individuals. The disease has been explored for its exact mechanism from all possible scales of molecular biology to deep insights of genetics. Various theories have covered long range of possible causes of cancer viz. cellular fluids, cellular events, tissue level modifications and even genetic aberrations [1–9]. Despite the fact that, different types of cancer differ in their primary causes, linked tissues, progression patterns and converging pathophysiology, there appears many overlapping features in common. These common features are accelerated cell division, altered, rewired and escalated metabolic pathways, [10, 11] distorted shape, abnormal nucleus, [12] inefficient mitochondria, acidic intracellular environment, contact inhibition, loss of apoptosis, angiogenesis, metastasis and many others. These common and overlapping features indicate an unidentified underlying common cause, which is although obvious, need some reflection.

In the last decade, carcinogenesis has been consistently proven to be an evolutionary process and thus it must have a purposeful cause [13]. This evolutionary paradigm begins with cellular environment, travels through biochemistry and finally codes out in terms of its genetics. Here, cellular biochemistry plays an amalgamating role between environment and genetics. In order to identify the exact cause and mechanism of cancer, the purpose (why) is more important than the cause (how). Present work connects the well-known and lesser known findings in cancer research to highlight the underlying transition route through which a normal and healthy cell supposedly transforms to its cancerous phenotype.

## Methodology

Cancer research has been exploring all possible dimensions to identify the exact causes of cancer. Present cancer hypothesis, the Nexus model, is an effort to encircle primary cancer causes, cellular biochemistry, epigenetics and genetics in a single model where each of them acts as a node in transition route. The Nexus model explains the purpose behind cancer evolution and so as the cause.

### The Nexus model

**Fig. 1:**
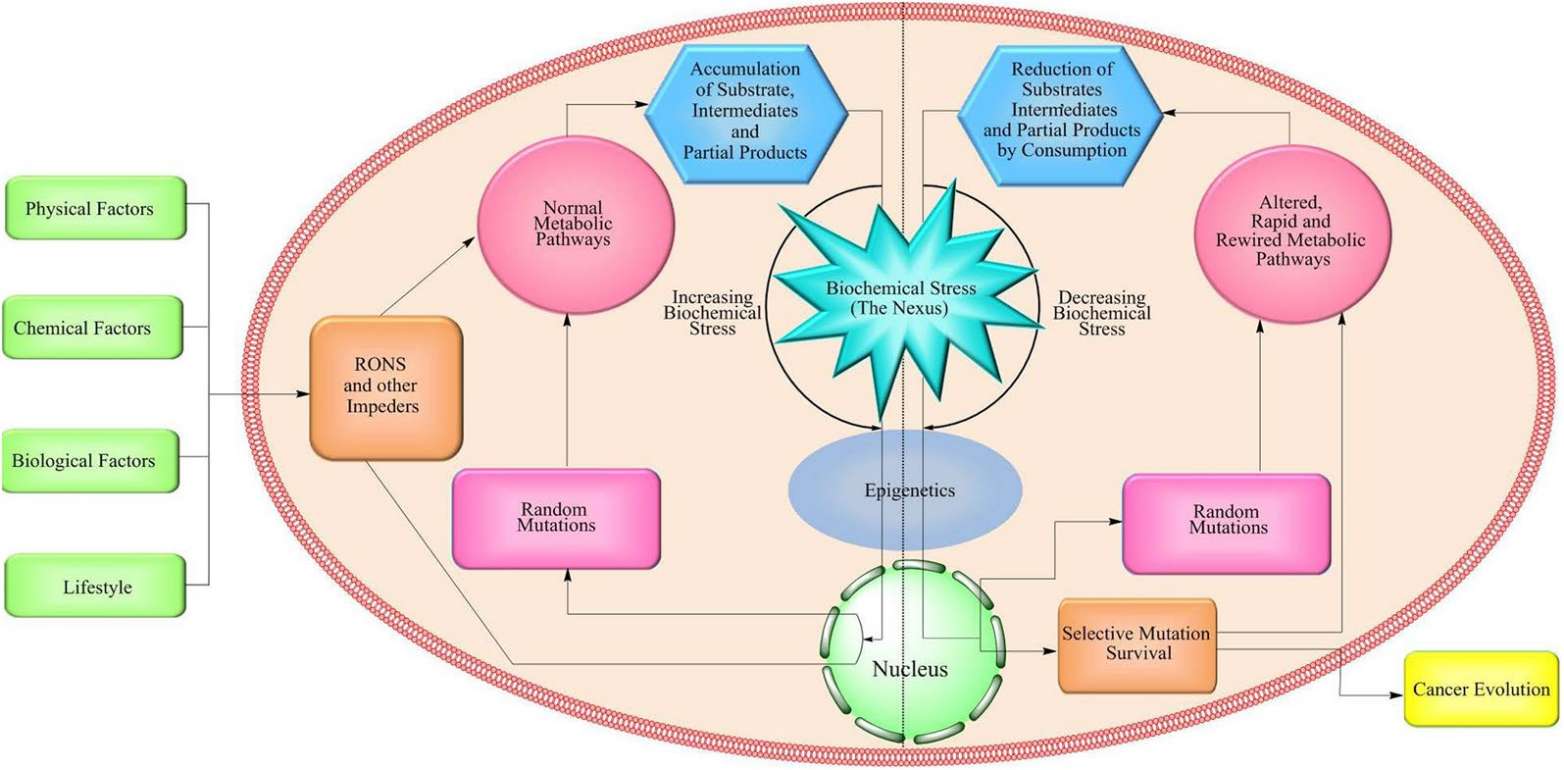
The overall diagram for the *Nexus* model representing the most probable transition route in cancer evolution

This model proposes that the probable transition route opens with the primary inducers (established primary causes) such as physical, chemical, biological and lifestyle related causes (Fig. 1). Such primary inducers then interact with the cellular biochemical pathways and generate reactive oxygen and nitrogen species (RONS) along with other free radicals, also known as cellular impeders (Fig. 1). The RONS, free radicals and viruses can also bring random genetic aberrations, which then generates structurally and functionally altered regulatory molecules (biomolecules) involved in metabolic pathways [14–16]. The interference of the cellular impeders thus results in the accumulation of initial substrates, intermediates or partial pathway products. Such an accumulation of the biologically insignificant metabolites congest cellular traffic thereby leading to a cellular environment that hampers the breakdown of normal metabolic pathways. This further develops an overload of residual metabolites in the cellular environment. Such a scenario results in the loss of intercellular signaling in a tissue and ultimately cause prolonged cellular biochemical stress that continues through many cell cycles, and eventually alters the cellular microenvironment. Such a complete alteration of the cellular microenvironment and the loss of intercellular signaling then creates perfect platform to initiate chain of events responsible for epigenetic and genetic changes [17]. Such events cause prolonged biochemical stress, thereby inducing considerable changes in stressed cells and marking the beginning of cellular events leading to cancer. Such events are hereby called as the Nexus. Initially, such mutations are random and result into expression of biomolecules which may either add to or reduce the biochemical stress (the Nexus) [18], better known as positive or negative cellular feedback. While the “positive feedback” refers to the survival of the mutations that reduces cellular stress, “negative feedback” refers to the mutations that might contribute to the increase of substrates, intermediates and partial products. The positive feedback is evidently repeated in the forthcoming cycles featuring mutational selection, resulting into newly evolved genetic machinery powered by such selective mutations [13, 18, 19]. On the other hand, such mutations are also capable of consuming residual metabolites via rewired metabolic pathways and have high rates of proliferation and turn out to be cancerous [20–27]. Over time, survival and accumulation of selective mutations which aid to positive feedback result in cancer phenotype (cancer evolution) of a healthy cell. The word ‘Nexus’ justifies the role of biochemical stress as the junction where strings originated from primary inducers further travel to epigenetics and genetics in cancer evolution.

In order to substantiate the Nexus Model, experimental and established evidences have been composed below under Phase I, Phase II and Phase III.

### Phase I: primary inducers and cellular impeders

**Fig. 2:**
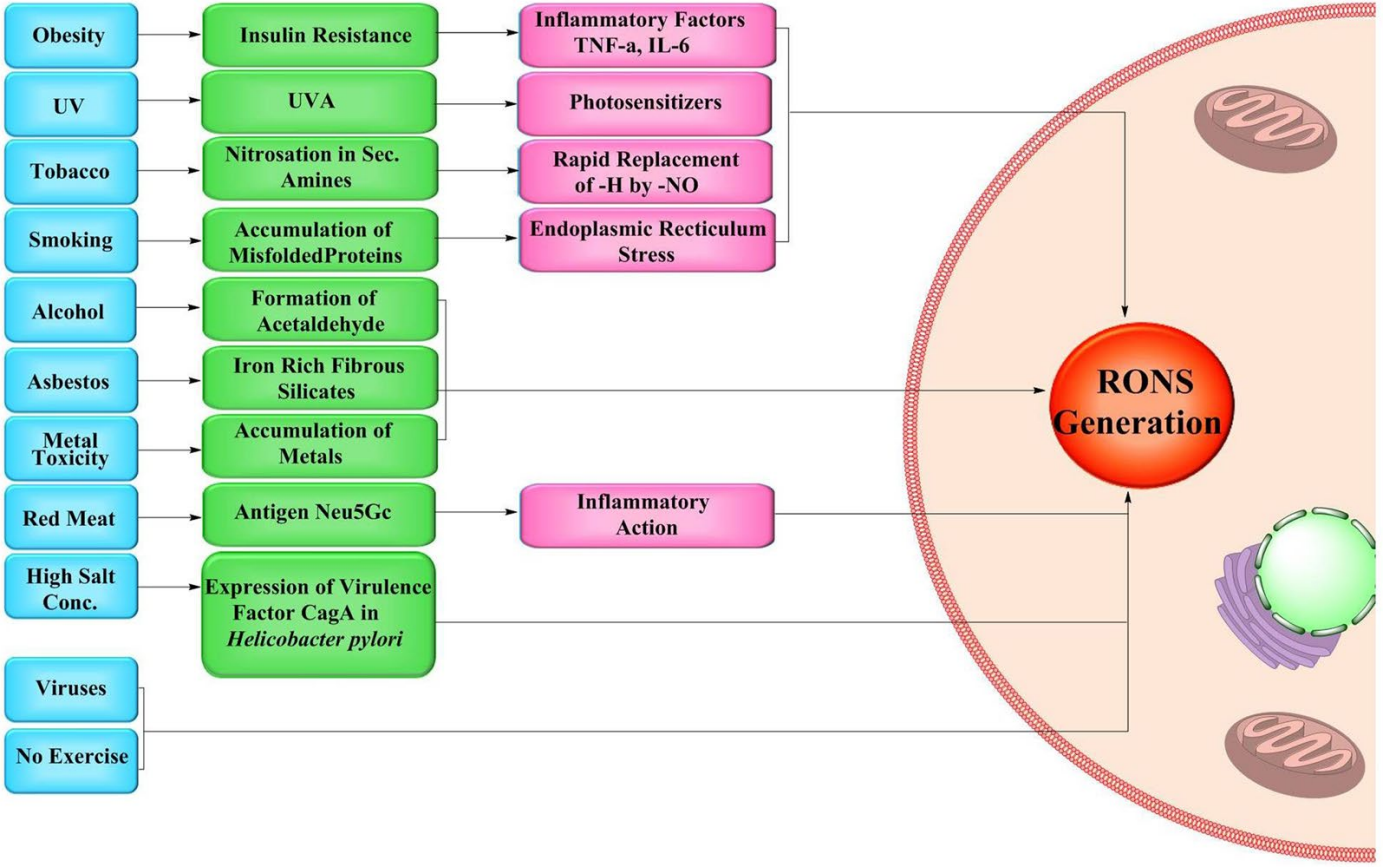
Phase I (The Nexus model): Primary cancer causes (physical, chemical, biological and lifestyle) and their sequential products to end up in form of RONS

Oxidative stress is a condition which results due to the production of oxidative radicals, mostly reactive oxygen species (ROS) and reactive nitrogen species (RNS) that exceeds the quenching limit of cells [28]. RONS can be generated by a number of inflammatory reactions, physical and chemical factors [28–30] (Fig. 2). It is an established fact that lifestyle factors, which include cigarette smoking, sun exposure, workplace, diet etc., affect the chances of acquiring cancer [31, 32].

According to the National Cancer Institute, obesity has been found to be prominently associated with the risk of cancer, wherein the United States alone, in 2012, 28,000 newer cancer cases in men and 72,000 newer cases in women were linked to obesity and being overweight [33]. Various factors released by the adipose tissues result in insulin resistance and consequent production of pro-inflammatory factors like tumor necrosis factor-α (TNF-α), interleukin-6 (IL-6) and cytokines which end up in unusual production of ROS [34, 35]. Another factor is UV radiation that has a prominent role in causing skin cancer [36]. Most of the Ultra Violet (UVA) energy is taken by the photosensitizers in the cells which are believed to generate ROS [37]. Tobacco, cigarette smoke, alcohol, naturally occurring fibrous substance-asbestos and metal toxicity are some chemical factors which are found to be responsible for the production of RONS. Tobacco contains nicotine and structurally similar alkaloids consisting of secondary and tertiary amines which react with nitrite forming nitrosamines [38]. In case of secondary amines, nitrosation is an exceptionally fast process, in which –H atom attached to nitrogen is replaced by –NO [39, 40]. The –NO group being a potential reactive species causes oxidative stress [41]. Exposures to smoke produced by cigarettes can be blamed for the oxidative stress as it persuades the aggregation of misfolded proteins and endoplasmic reticulum (ER) stress and consequently enhances the production of ROS [42–46]. Ethyl alcohol is converted into acetaldehyde in the body which is a budding cause of ROS production in the cells [47, 48]. Asbestos fibers are known to induce the cells to produce ROS due to the iron present on the fibrous silicates [49, 50]. Exposure to lethal waste sites, mines and construction sites may also subject the workers to high intensity metal toxicity of mercury, lead, arsenic etc. [51, 52]. Accumulation of these metals can then lead to the generation of ROS in cells.

Red meat, high salt consumption, viral infections and physical inactivity encompass the biological factors. Antibodies are produced in response to Glycolylneuraminic acid [Neu5Gc], which acts as an antigen to the body, present in red meat. This interaction results in ignition of inflammatory cells thus producing ROS [53]. High salt consumption is also found to be a potential cause of ROS production [54]. The increased salt concentrations alter the expression of virulence factor CagA (cytotoxin-associated gene A) in Helicobacter pylori strain 26695, which is a highly acknowledged factor for cancer [55]. The infections due to *Human papillomavirus* (HPV) cause oxidative stress which in turn damage the cell DNA [56, 57]. Exercise is found to decrease the ROS production in body [58].

The above discussed factors summarize how physical, chemical, biological and lifestyle related factors, termed as primary inducers (primary causes) generate RONS and other cellular impeders that hamper cellular metabolic pathways. Such interference then causes accumulation of cellular substrates that eventually converge to forcibly induce biochemical stress thereby facilitating the evolution of cancer. The diagrammatic illustration of the same has been provided in Fig. 2.

### Phase II: development of biochemical stress (The Nexus)

Generation of reactive oxygen and nitrogen species (RONS) in the cells cause havoc in the normal functioning of enzymes and other biomolecules participating in various metabolic pathways [32]. At normal levels of RONS, the combat mechanisms are capable enough to maintain homeostasis inside cells, but when their concentration exceeds the threshold level, they impede the normal functioning inside the cell. High concentration of RONS interferes or reacts to cause delay, halt or even total loss in integrated framework of metabolic pathways [59, 60]. As in glycolysis, the elevated concentration of RONS oxidizes and thus inactivates pyruvate kinase monomer 2 (PKM2) which is responsible for the conversion of phosphoenol pyruvate (PEP) to pyruvate [54]. Similarly, high levels of RONS regulate the Hypoxia-Inducible Factor-1 (HIF1) to create hypoxic conditions, which is one of the most common features recorded in almost all type of cancer cells [61]. The HIF modulates the activity of pyruvate dehydrogenase kinase 1 (PDK1) thereby restricting the activity of pyruvate dehydrogenase (PDH) which prevents the conversion of pyruvate into acetyl CoA, hence cause hindrance in the tricarboxylic acid cycle (TCA) [62–64]. Obstruction in the TCA cycle greatly reduces the production of ATP via electron transport chain (ETC) [62]. To maintain the redox homeostasis, glycolysis adopts to pentose phosphate pathway (PPP) which is the principal pathway for de novo synthesis of nucleotides and this shunting of pathway generates excess of nucleotides [63]. Generation of excess nucleotides through PPP pathway causes substrate accumulation in cytoplasm. Generation of excess nucleotides through PPP pathway causes substrate accumulation in cytoplasm. Partial or complete obstruction of glycolysis [65] leads to the accumulation of substrates which were supposed to be consumed under normal conditions [66]. As all the metabolic pathways are interdependent [67], it is safe to say that hindrance in a single pathway leads to the upheaval in the other linked or parallel pathways.

The HIF-1 also triggers the activity of hypoxia inducible factor 2 (HIF-2) which causes amassing of lipids in the form of droplets [68] and simultaneous loss of expression of Von Hippel-Lindau gene (VHL) [69]. The loss of VHL expression further leads to the reduced expression of β-oxidation genes causing the curtailment of the β-oxidation pathway [70]. The reduction in the pathway eventually results in the accumulation of lipids. Nevertheless, de novo lipogenesis continues by using other carbon sources such as acetate and glutamine. The de novo synthesis is mediated by an increased level of fatty acid synthase (FASN) [71, 72].

Apart from the discussed routes that affect the biochemical pathways, RONS can cause direct damage to the DNA thereby causing random mutations [73]. These random mutations may occur in the genes that code the enzymes involved in the cellular metabolism and again lead to the disruption of these pathways, ultimately causing accumulation and biochemical stress. One such example is of isocitrate dehydrogenase (IDH) mutation. IDH is an enzyme which catalyzes conversion of isocitrate into α-ketoglutarate [74] and provides defense against oxidative insults. Genetic alteration in the IDH gene results in the alteration of its enzymatic activity. This mutated form of the enzyme catalyzes the conversion of α-ketoglutarate into 2 hydroxyglutarate (2-HG) which is a well-known oncometabolite [75, 76]. High concentration of the 2-HG thereby results in abnormal DNA hypermethylation in cells [77].

Another metabolite that is involved in creating the biochemical stress (Nexus) is the enzyme fumarate hydratase (FH). The RONS guided mutations inactivate FH, which leads to the blockage of TCA cycle [78, 79]. It has two possible consequences; first, it causes accumulation of fumarate and succinate leading to biochemical stress; second, the accumulated fumarate reacts with reduced glutathione (GSH) producing succinated glutathione which is considered to be an oncometabolite [78]. This oncometabolite is further degraded by glutathione reductase releasing GSH which then combines with fumarate in an aborting manner consuming NADPH, ultimately obstructing RONS detoxifying potential of the mitochondria and thereby increasing the RONS generation [79]. Eventually, it leads to substrate accumulation and hence elevated biochemical stress (The Nexus) [80].

**Fig. 3:**
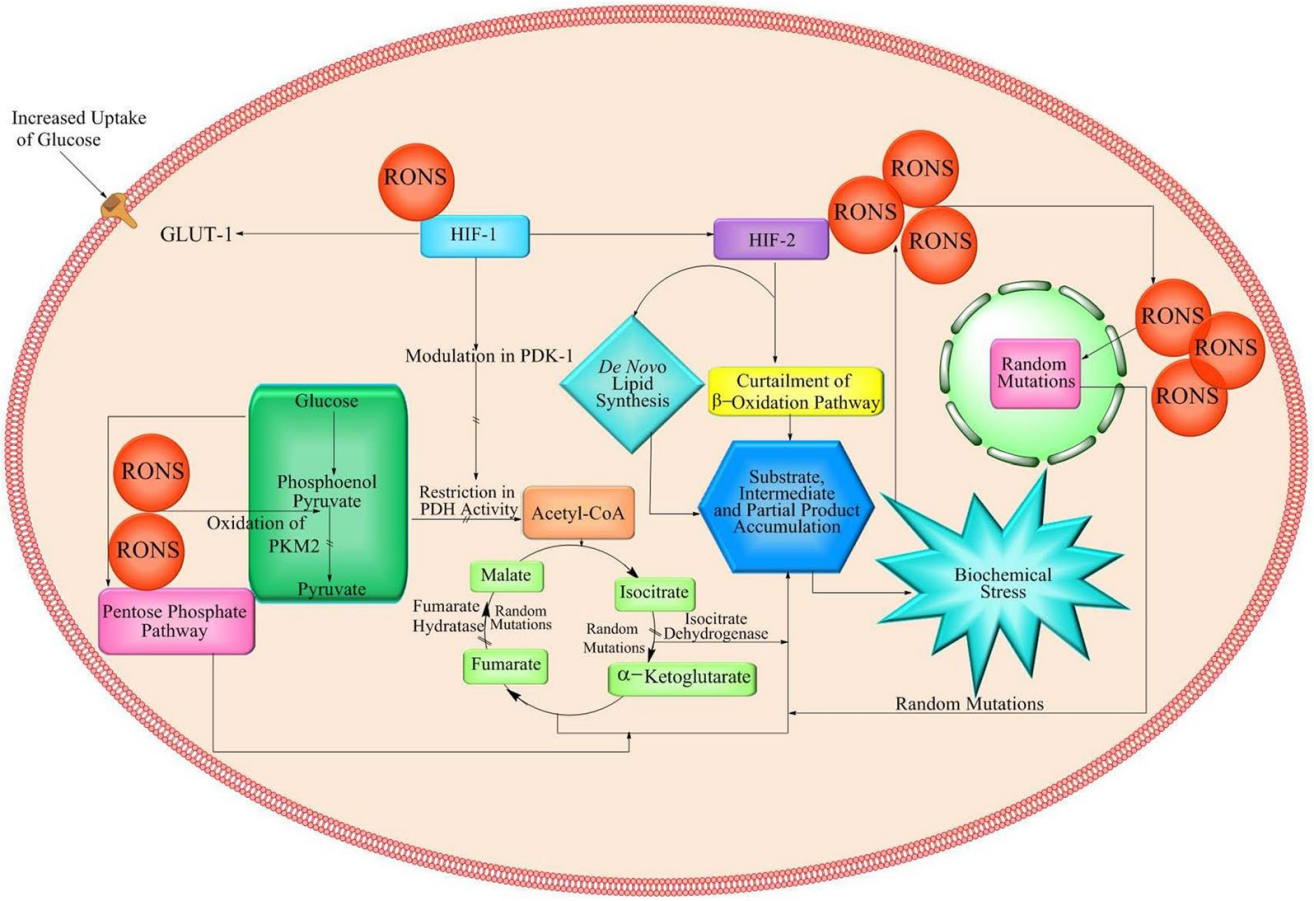
Phase II (The *Nexus* model): RONS and their interference leading to development of biochemical stress i.e. The *Nexus*

It can be deduced from the above-discussed facts that high RONS concentration resulting from primary inducers can, directly and indirectly, affect the normal cellular metabolic pathways. Any type of delays, layoffs or outright loss in any of the metabolic pathways results in the accumulation of substrates, intermediates and partial products. Accumulation of these components then enhances cellular traffic resulting into an overflowing abundance of such products inside the cytoplasm. Under such a condition where nutrients are no more consumed efficiently, the machinery of bioenergetics (ATP) starts to shutdown and the futile intermediates and partial products simultaneously increase the cellular traffic to generate biochemical stress (The Nexus). The prolonged biochemical stress cuts off the intercellular signaling in the affected tissues thereby bringing the cells into isolation. Cellular isolation and long term biochemical stress can be considered as the perfect conditions to stimulate epigenetics followed by genetic evolution. The above enlisted events can be considered as phase II of cellular transition in cancer described in Fig. 3.

### Phase III: epigenetics to genetic evolution

#### Biochemical stress to epigenetics

Long term biochemical stress and interrupted intercellular signals in linked tissues create a new microenvironment which further acts as a driving signal that prepares cells for genetic evolution for biochemical negotiation. These driving signals are epigenetic changes which result in abnormal gene functions and aberrant patterns of gene expression and are usually observed in all types of cancer. Growing evidences suggest that the acquired epigenetic abnormalities interact with genetic alterations over time to cause dysregulation in the routine functioning of cells [81]. Few of the supportive findings have been produced and discussed below which collectively explain direct or indirect effect of RONS and consequent biochemical stress on cellular epigenetics and genetics.

Epigenetics involves endowment of instructions based on the expression of genes. The major modifications that basically comprise epigenetic changes are methylation, acetylation and phosphorylation which results in post translational histone modifications [82].

The prolonged exposure of the tissues to this RONS driven biochemical stress (The *Nexus*) and other environmental factors bring about epigenetic changes which marks the initiation of phase III in cellular transition. The stressful environment then generates several types of responses to combat the stress, most of which lead to epigenetic alterations. It is known that oxidative stress causes accumulation of unfolded proteins in ER, activating unfolded protein response (UFR) by altering the levels of molecular chaperone GRP78/BiP (78 kDa glucose-regulated protein/binding immunoglobulin protein), a master regulator of ER functions and contributor of tumor cell survival and growth [83, 84].

Stress proteins like heat shock proteins mediate an increase in chaperone protein activity which enhances protein folding capacity, thus counteracting stress and promoting cell survival [85]. DNA lesions caused as a result of oxidative stress are genotoxic and also prompt genetic mutations [86]. RONS have also found to be interfering with the cell death mechanisms, either acting as an anti-senescence agent or through the specific stimulation of AIF (apoptosis-inducing factor). It helps in suppressing apoptosis and therefore maintains the phenotypic transformation of cancer cells [87]. A recent study has showed that various oxidized products (dimethyl and methionine sulfoxide) may accumulate in the cytosol during the initial stages of carcinogenesis and react with nearby nucleotides, leading to aberrant methylation-induced gene silencing [88]. All these reports confirm that biochemical stress as the *Nexus* creates necessity and acts as a source code for epigenetic makeup during cellular transition in cancer.

#### Cancer epigenetics

Cellular transition further continues when these epigenetic abnormalities lead to disturbances in the cellular genetic makeup [89]. Epidermal growth factor receptor (EGFR) is one such example which governs the signaling pathways involved in the regulation of growth, metabolism, differentiation and apoptosis under stressed conditions through its tyrosine kinase (TK) activity. Mutation in the epidermal growth factor receptor-tyrosine kinase (EGFR-TK) domain in ovarian cancer has resulted in the over production of EGFR [90, 91]. This overproduction in turn alters the activity of DNA methyltransferase, an enzyme which is responsible for DNA methylation [92–95]. DNA methylation is the most widely investigated epigenetic modulation in cancer. In normal conditions, it regulates gene expression and inactivation. The methyl group covalently attaches to the cytosine residues in the CpG dinucleotides [96, 97]. These CpG sites are not randomly distributed in the genome; instead the CpG rich regions are known as CpG islands and they generally cluster at the 5′ end of the regulatory region (generally the promoter region) of many genes [89, 96]. These islands are not methylated in normal cells [98]. Hypermethylation of CpG islands in the promoter region of tumor suppressor genes is a major event in the origin of many types of cancer. Hypermethylation of promoter region of CpG-islands out-turns into complete or partial loss of genes involved in the normal functioning of cell including those involved in cell cycle, DNA repair, and metabolism of carcinogens, cell to cell interaction, apoptosis and angiogenesis [96]. The methylated CpG islands are not capable of initiating transcription and hence there is an altered gene function. Thus, hypermethylation at the promoter region favors the mechanism of mutation and helps to accelerate random mutations during cellular transformation [99]. As a result of hypermethylation in the promoter region, the tumor suppressor gene p16, which regulates the proliferation rate of the cell is not transcribed and thus gets inactivated. Inactivation of gene p16 leads to the uncontrolled proliferation of tumor cells [100]. Mutations take place in the mTOR (mammalian Target of Rapamycin) signaling pathway due to the loss of tumor suppressors or activation of oncogenes promotes tumor growth and survival. Activation of mTOR pathway is also reported to take place under stressed conditions such as nutrient deprivation [10, 101]. Reports which have identified hypermethylation of many genes in various cancers are collectively presented in Table 1.

**Table 1:** Hypermethylation of various genes investigated in different types of cancers

S. N.	Site of hypermethylation	Type of cancer
1	Glutathione S transferase gene (GSTPI, also known as GST3)	Prostate and breast cancer [91]
2	Promoter region of Liver Kinase B1 (LKB1)	Papillary breast cancer [92]
3	Promoter region of cyclin-dependent Kinase inhibitor p15INK4B	Leukemia and glioma [93]
4	Promoter CpG island of the O6-MGMT gene	Brain, colon and lung cancer [94]
5	CpG island of death-associated protein Kinase DAP-Kinase	Lymphomas [95]
6	Promoter region of p73 gene	Lymphoma cancer [96]

Apart from DNA methylation, there are other covalent modifications like histone modifications which control gene activity and play a major role in cancer development [100]. Post translational histone modifications have direct influence on chromatin structure and function. It usually results in rewired gene regulation; it includes histone deacetylation and histone acetylation [102]. Histone acetylation mediated by histone acetyltransferase (HAT) and histone deacetylation mediated by histone deacetylase (HDAC) plays a crucial role in gene expression and silencing. HDAC is found to be related with tumor development as it induces transcriptional inactivation [10, 103]. The deacetylation of lysine residues of histone 3 and histone 4 largely increases the ionic contact between positively charged DNA packaging proteins (histone) and negatively charged DNA which condenses the chromatin and makes transcriptional gene inert [104, 105].

Transcriptional blockage of tumor suppressor gene by upregulation or bizarre recruitment of HDACs to their promoter site is a common feature for emergence and tumor development [106]. The acetylation status of histones H3 and H4 seem to largely dictate the fate of chromatin assembly, transcription, and gene expression [107, 108]. Histone acetylation is governed by the opposing activities of HATs and HDACs [109, 110]. Thus the loss of normal functioning of gene opens a divergent pathway to escape early senescence, leading to genetic changes, which results in the escape of cancer cells from apoptosis [111].

**Fig. 4:**
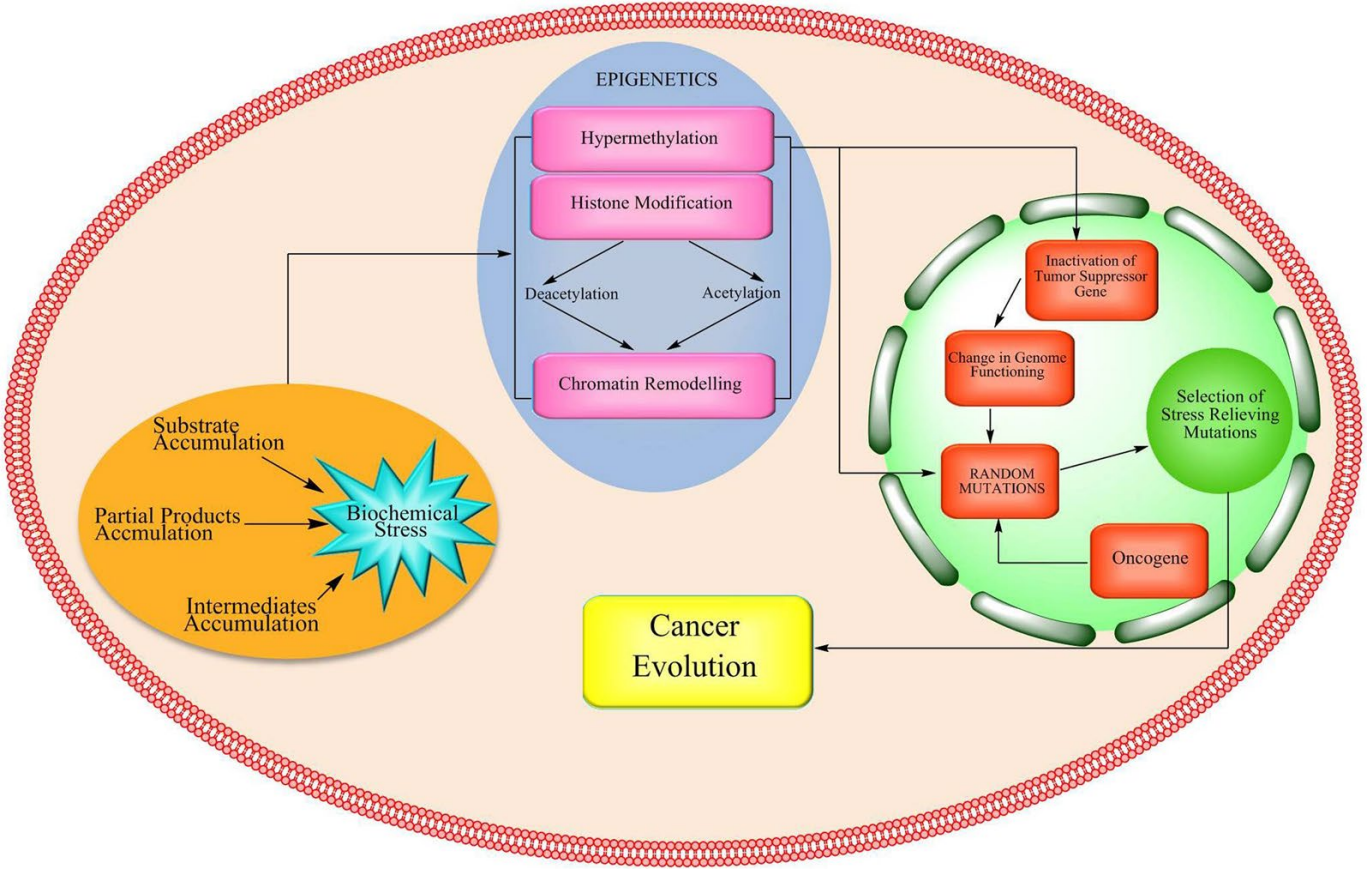
Phase III—Biochemical stress (The *Nexus*) to epigenetics and genetics in cancer evolution

The above evidences conclude that biochemical stress (the *Nexus*) induces epigenetic events which helps cell to rule out normal gene expression and create a demand of improved genetic makeup which could off load the prolonged biochemical stress. Once these epigenetic alterations mediate genetic changes, the last step in the phase of cellular transition of selection, adaptation and evolution comes into play. These events are depicted in Fig. 4.

#### Cancer genetics

The loss of functional genes by epigenetic silencing has been shown to mediate genetic mutations leading to the development of tumor cells [112]. The epigenetic alterations initiate a cascade of reactions which may not only shut a single pathway but also affect other important signaling pathways. Further these abnormalities conduct linked distortion of metabolic pathways to promote tumorigenesis [113]. Loss of intercellular signals resulted from biochemical stress acts as a catalyst in genetic evolution. Initially, the genetic mutations are random and may occur as a genetic response to epigenetic codes developed from prolonged biochemical stress. Progressively, mutations which help the cell to overcome residual content, promote the errant growth and help to relieve the cellular stress are selected naturally. Stress inducible mutagenesis mechanism can potentially accelerate adaptive evolution of cancerous cells. A few examples supporting the selective adaptation and evolution have been collected here.

In many colon cancers, a mutation that inactivates the tumor-suppressor gene called APC (adenomatous polyposis coli) is the first or at least a very early, step in cancer progression. APC mutations can be detected in small benign polyps at the same high frequency as in large malignant tumors, suggesting that they occur early in the process. The loss of APC activity gives the affected cell a growth advantage, allowing it to form a colony of cells that divide more rapidly than they die. The increased proliferation leads to the growth of a polyp [114], pointing out the possibility of the fact that a particular mutation gets selected only when proved beneficial in evolution inside the cell. Once cells lose their ability to repair these replication errors, mutations can accumulate in many genes, including tumor suppressor genes and oncogenes. Patients with this genetic defect develop one or two tumors that then progress rapidly to a full-blown cancer [115].

The breast cancer genes (*BRCA1* and *BRCA2)* are found to mediate DNA damage control in cells and regulation of transcription. Mutations in these two genes are profoundly associated with occurrence of breast cancer and ovarian cancer. It has been evaluated and confirmed in vitro that absence or mutations in these genes result in uncontrolled proliferation and tumor development [116]. Similarly, mutations in epidermal growth factor receptor (EGFR) gene have been identified in lung adenocarcinomas helping cancer cells in proliferation, migration and metastasis [117]. Many other gene mutations are strongly linked to numerous cancers; these mutations support common features of cancer cells. The altered functions of these mutated genes in cancer appear to be beneficial in evolution. The cancer evolution has a purpose where selective mutations act as soldiers to fight against cellular biochemical stress via altering, accelerating or rewiring cellular processes so as to reduce nutritional metabolite overload and accumulation resulted from cellular impeders.

## Results and discussion

The ‘*Nexus*’ model connects primary cancer-causing factors, cellular biochemistry, epigenetics and genetics in cancer. By naming the epicenter of all such events as the ‘*Nexus’*, we have tried to justify the purpose on which a healthy cell under stress persuades to transform to its cancer phenotype. This model may add a new dimension and perspective to cancer research where to understand the exact cause of cancer; we must first discover the purpose of evolution. The questions to be addressed should be, why cells choose to evolve or transform to cancerous form and in what context the evolution is beneficial to the cell. The *Nexus* model would lead to finding new drug targets which are directly or indirectly involved in accumulation of metabolites and add or reduce biochemical stress in a cell. The unfathomable queries linked to cancer may be answered using the *Nexus* model.

This model links primary causes to cancer development but not directly. It validates usual enquiry of why all people exposed to primary causes of cancers (e.g. alcohol, tobacco) do not develop cancer whereas individuals who are not at all exposed to any one of the primary causes show linked cancer incidences. There could be ancillary possible reasons which may cause biochemical stress other than primary inducers in linked tissues. Over a past few centuries, many theories on cancer development have been proposed. The *Nexus* model encompasses and validates such major preexistent theories viz. trauma theory, infectious disease theory, somatic mutation theory, tissue organization field theory and epigenetic theory. These theories and their indicated causes could be linked to biochemical stress in a way or other way around.

## Conclusions

Common features of cancer cells imply towards a common underlying cause of cancer irrespective of their origin and pathophysiology. Primary causes are not directly linked to cancer evolution; rather, they end up with the production of cellular impeders (RONS). Perpetuated biochemical stress resulted from the accumulation of substrates, intermediates and partial products acts as ‘The *Nexus*’. The *Nexus* is the end product of primary inducers and cellular impeders. It develops altered cellular environment which acts as a key ingredient of cancer epigenetics. The codes retrieved from ‘The *Nexus*’ are processed by epigenetics and are finally forwarded to cancer genetics. At first, the mutations are random but become selective when they help the cell to overcome the biochemical stress. Selective mutations are found to outlaw normal cellular processes, promote accelerated and aberrant growth, and rewire metabolic pathways and many other common benefits to negotiate with extended biochemical stress. The *Nexus* may act as the switch and the common cause in cancer evolution.

## Abbreviations

RONS: reactive oxygen and nitrogen species
ROS: reactive oxygen species
RNS: reactive nitrogen species
BMI: body mass index
TNF-a: tumor necrosis factor-a
IL-6: interleukin-6
UVA: ultra violet A
ER: endoplasmic reticulum
Neu5Gc: N-glycolylneuraminic acid
CagA: cytotoxin-associated gene A
HPV: human papillomavirus
PKM2: pyruvate kinase monomer 2
PEP: phosphoenol pyruvate
HIF-1: hypoxia inducible factor-1
GLUT-1: glucose transporter-1
PDK1: pyruvate dehydrogenase kinase 1
PDH: pyruvate dehydrogenase
TCA: tricarboxylic acid cycle
ETC: electron transport chain
PPP: pentose phosphate pathway
HIF-2: hypoxia inducible factor-2
VHL: Von Hippel-Lindau gene
FASN: fatty acid synthase
IDH: isocitrate dehydrogenase
2-HG: 2-hydroxyglutarate
FH: fumarate hydratase
GSH: glutathione
UFR: unfolded protein response
BiP: binding immunoglobulin protein
GRP78: 78 kDa glucose-regulated protein
AIF: apoptosis inducing factor
EGFR: epidermal growth factor receptor
EGFR-TK: epidermal growth factor receptor-tyrosine kinase domain
mTOR: mammalian target of rapamycin
PI3K: phosphatidylinositol-4,5-bisphosphate 3-kinase
AMPK: 5′ AMP-activated protein kinase
HAT: histone acetyltransfarase
HDAC: histone deacetylase
APC: adenomatous polyposis coli
BRCA1: breast cancer 1
BRCA2: breast cancer 2
